# Anatomical type analysis of right interlobar artery based on chest thin-slice CT scan and three-dimensional reconstruction

**DOI:** 10.1186/s13019-022-02088-y

**Published:** 2022-12-20

**Authors:** Long-fei Wang, Lei Zhao, Chang-sheng Lv, Qing-tao Xu, Rong Wang

**Affiliations:** 1 Ningbo First Hospital, Ningbo, 315000 China; 2 The First Affiliated Hospital of Dalian Medical University, Dalian, 116000 China; 3 Meizhou City People’s Hospital, Meizhou, 514000 China

**Keywords:** Three-dimensional reconstruction, Computed tomography, Pulmonary artery, Early non-small cell lung cancer, Anatomy

## Abstract

**Purpose:**

To analyse and summarize branching pattern types of the interlobar portion of right pulmonary arteries (RPA) through chest thin-slice CT scans and three-dimensional reconstruction.

**Methods:**

A total of 179 patients (58 males and 121 females, with an average age of 53.9 years) at the Thoracic Surgery Department of Ningbo First Hospital were retrospectively included from December 2020 to December 2021. All patients completed preoperative thin-slice CT scans and three-dimensional reconstructions of the chest. The clinical data and branching patterns were collected. Data were analysed using SPSS 21.0.

**Results:**

The branching pattern types of the interlobar portion of RPA were divided into 4 types according to the order and number of branches: Type I (145/179, 81.0%), Asc. A2, MA, A6; Type II (28/179, 15.6%), Asc. A2 deletion, MA, A6; Type III (5/179, 2.8%), Asc. A2, A6, MA; and Type IV (1/179, 0.6%), MA, Asc. A2, A6. Type I was the most common pattern. Furthermore, according to the number of branches of MA and A6, this pattern can be subdivided into 15 subcategories.

**Conclusion:**

Chest thin-slice CT scans and 3D reconstructions can provide surgeons with accurate lung anatomy, which helps surgeons perform preoperative planning and complete surgery successfully.

## Introduction

With the prevalence of low-dose CT scanning [1], early-stage lung cancers are more frequently discovered. Both the National Comprehensive Cancer Network (NCCN) guidelines and the Chinese Society of Clinical Oncology (CSCO) guidelines recommend early local surgical resection as the first choice for the treatment of early-stage non-small cell lung cancer. Surgical resection has changed from pneumonectomy to lobectomy [2], and lobectomy is still considered to be the standard approach for lung cancer. However, with changes in the types of diseases, multiple studies have confirmed [3, 4] that video-assisted thoracoscopic surgery (VATS) anatomical sublobar resections (e.g., segmentectomy, subsegmentectomy, and combined segmentectomy/subsegmentectomy, etc.) does not result in significant differences in perioperative mortality and complication rates for patients with early-stage non-small cell lung cancer compared with traditional lobectomy. However, these approaches yielded better 5-year survival and better lung function protection. For early-stage non-small cell lung cancer, segmentectomy may replace traditional lobectomy as the standard procedure in the near future.

Surgeons need to be proficient with the anatomy of lung segments and subsegments, especially for rare vascular and bronchial variations. Although thin-slice CT scans provide thoracic surgeons with anatomical structures, they require a long learning curve [5]. In other words, the anatomical variations and complexity of the right pulmonary artery, while easily understood by a radiologist, are more readily understandable to the surgeon in a 3D format. Several studies have shown that preoperative 3D reconstruction [6–9] is useful in accurately identifying the branching patterns of pulmonary arteries, shortening the operation time and reducing the incidence of complications, such as intraoperative bleeding and conversion to thoracotomy. In addition, inexperienced surgeons require a learning curve of 30 cases for 3D reconstruction-assisted VATS anatomical sublobar resections. Although previous studies [10–15] summarized the common anatomical types of segmental bronchi and blood vessels through 3D reconstruction to allow surgeons to learn the relevant anatomy and improve preoperative planning, descriptions of the branching patterns of the interlobar portion of right pulmonary arteries were lacking. Therefore, in this study, the common types and rare variants of the interlobar artery in the right lobe were classified and summarized using thin-slice CT scans combined with 3D reconstruction.

## Materials and methods

### Patients

**Fig. 1:**
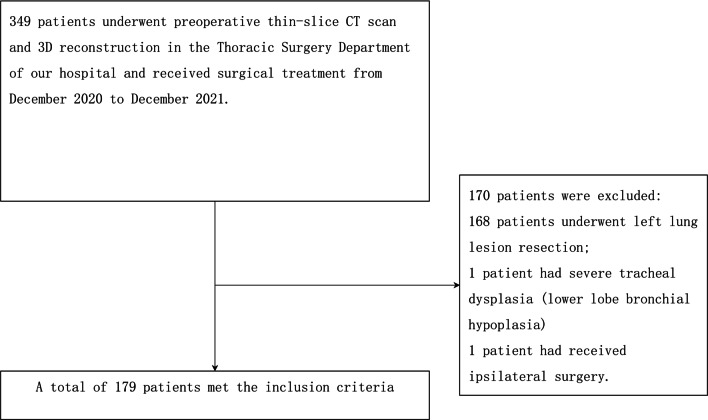
A total of 179 patients were included in the study

This study was approved by the Ethics Committee of Ningbo First Hospital. From December 2020 to December 2021, the clinical data of 349 patients who underwent thoracic thin-slice CT scans and 3D reconstructions and received surgical treatment in the Thoracic Surgery Department of Ningbo First Hospital were retrospectively collected. A total of 168 patients who received left lung lesion resection, 1 case of severe tracheal dysplasia (lower lobe bronchial hypoplasia), and 1 case of local recurrence after right posterior segmentectomy were excluded, and a total of 179 patients were included in this study (Fig. 1). Inclusion criteria: (1) Patients who had undergone surgical resection; (2) Patients with thin-slice chest CT scans and three-dimensional reconstruction; and (3) Patients with complete clinical data; Exclusion criteria: (1) Patients who underwent left pneumonectomy; (2) Patients who had ipsilateral surgery previously; and (3) Missing clinical data.

### Thin-slice CT scan and 3D reconstruction

All patients used the same CT machine to complete a thin-slice CT scan of the chest at the end of inspiration before the operation (scanning parameters: tube voltage 120 kV, tube current 200–300 mA, slice thickness 0.625–1.25 mm, slice interval 1 mm). 3D reconstruction software (InferVisual Surgery Planning, V2.0) was then used to create 3D images based on the imaging data of thin-slice CT plain scans. After creating a 3D reconstruction, we assessed 3D images using thin slice axial images with the assistance of cross positioning lines, through which pulmonary arteries can be located simultaneously on both thin slice axial images and 3D images.

### Anatomy of interlobar arteries

In the present study, the nomenclature of the pulmonary arteries (PA) is based on the Illustrated Anatomical Segmentectomy for Lung Cancer, which was edited by Hiroaki Nomori and Morihito Okada. Hiroaki Nomori described a prevailing pattern schema of the right pulmonary arteries: the first branch (A1 + A3 + A2a) arises from the anterior portion of the main right PA (RPA); the second branch (Asc. A2, the posterior ascending artery) was found on the posterolateral surface of the main RPA; the third branch (middle lobe artery, MA) arises anteriorly from the interlobar portion of the RPA; the fourth branch (A6) supplies the superior segment of the lower lobe. The interlobar portion of RPA is defined as branches as follows: Asc. A2, MA, and A6. The branching patterns of the main RPA of the interlobar portion were classified in the order of bifurcation from proximal to distal. The number and frequency of each type were recorded. When two blood vessels supplying different pulmonary segments were at the same positions, the branching patterns were based on the following principles: branching patterns were defined as Asc. A2, MA for cases with the same positions of Asc. A2 and MA; for cases with the same positions of MA and A6, the branching patterns were classified as MA, A6. When several branches of MA and A6 were observed, the position of the most proximal branch was regarded as the branch location. All cases were identified and classified by a thoracic surgeon and a radiologist. A conclusion was reached after discussion based on a thin-slice CT scan of the chest in cases of a dispute.

### Data analysis

The clinical data (age, sex, surgical technique, intraoperative and postoperative complications, postoperative pathology, etc.) and branching patterns of interlobar portion arteries were collected. Numerical data are expressed as the mean (range), and categorical data are expressed as frequencies (percentages). All data were statistically analysed using SPSS software version 21.0 (IBM, USA).

## Results

### Clinical data

A total of 179 patients [58 males and 121 females, average age 53.9 years (22–83 years)] were included in this study. Forty-three (24.0%) patients underwent lobectomy, 80 (44.7%) patients underwent segmentectomy, 19 (10.6%) patients underwent wedge resection, and 37 (20.7%) patients underwent more than two of the above surgery types. Pathology confirmed 162 cases (90.5%) of adenocarcinoma, 2 cases (1.1%) of squamous cell carcinoma, 1 case (0.6%) of small cell lung cancer, 12 cases (6.7%) of benign lesions, and 2 cases of metastasis due to another tumour. Only one patient experienced haemorrhage during surgery due to lymph nodes in our study (Table 1).

**Table 1:** Clinical data of 179 patients

Clinical characteristic	Mean (range)/frequency (frequency)
Age	53.9 (22–83)
Female	121 (67.6%)
Surgical methods	
Lobectomy	43 (24.0%)
Segmentectomy	80 (44.7%)
Wedge resection	19 (10.6%)
Combining two or more	37 (20.7%)
Postoperative pathology	
Adenocarcinoma	152 (84.9%)
Squamous cell Carcinoma	2 (1.1%)
Small Cell Lung Cancer	1 (0.6%)
Benign lesions	22 (12.3%)
Metastases from other sites	2 (1.1%)
Intraoperative complications	
Bleeding	1 (0.6%)
Transition thoracotomy	0 (0.0%)

(Combining two or more surgical procedures including lobectomy + segmentectomy, lobectomy + wedge resection, lobectomy + segmentectomy + wedge resection)

### The RPA branching patterns of the interlobar portion

**Fig. 2:**
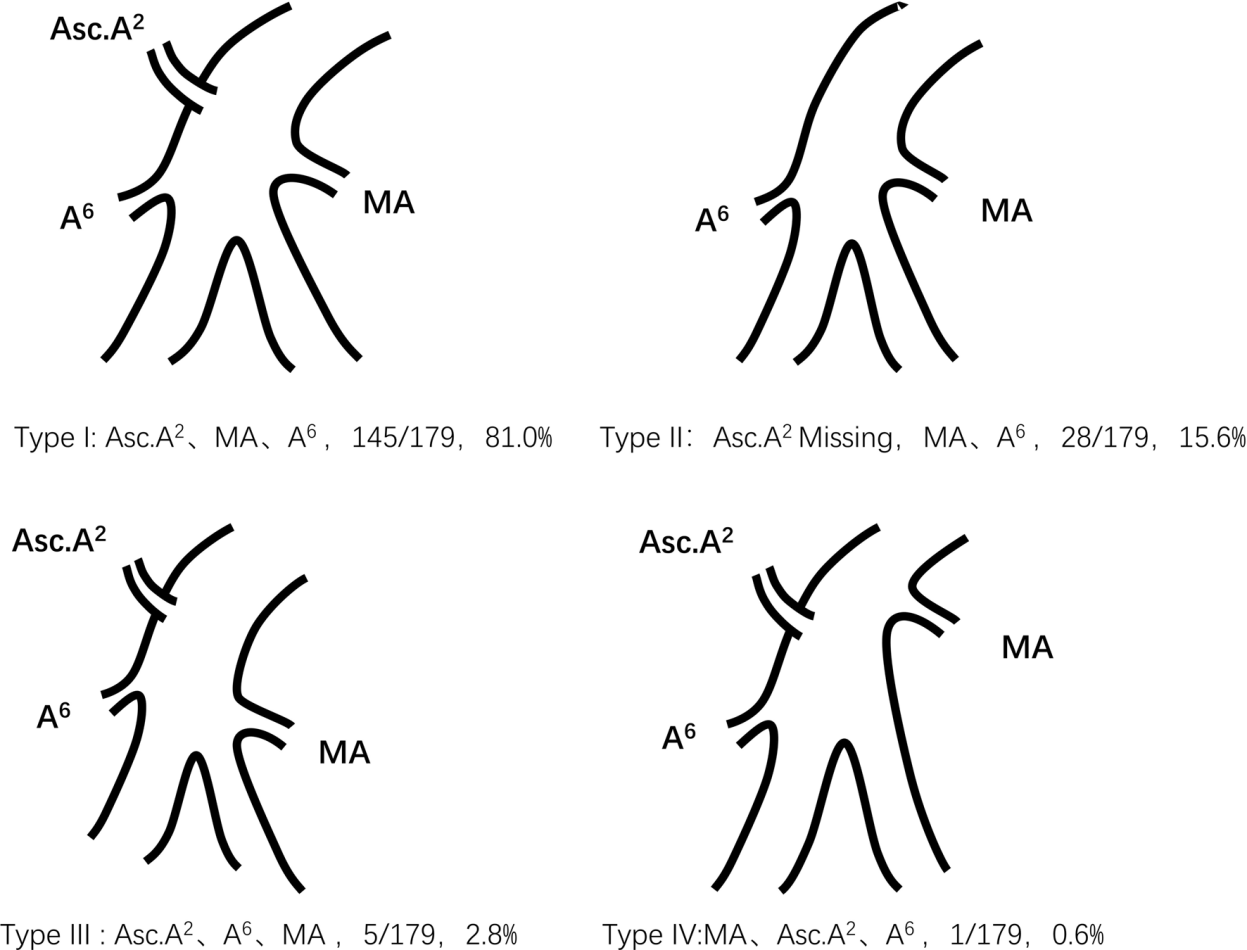
The RPA branching patterns of interlobar portion (RPA: right pulmonary arteries, MA: middle lobe artery, Asc.A2: posterior ascending artery)

**Fig. 3:**
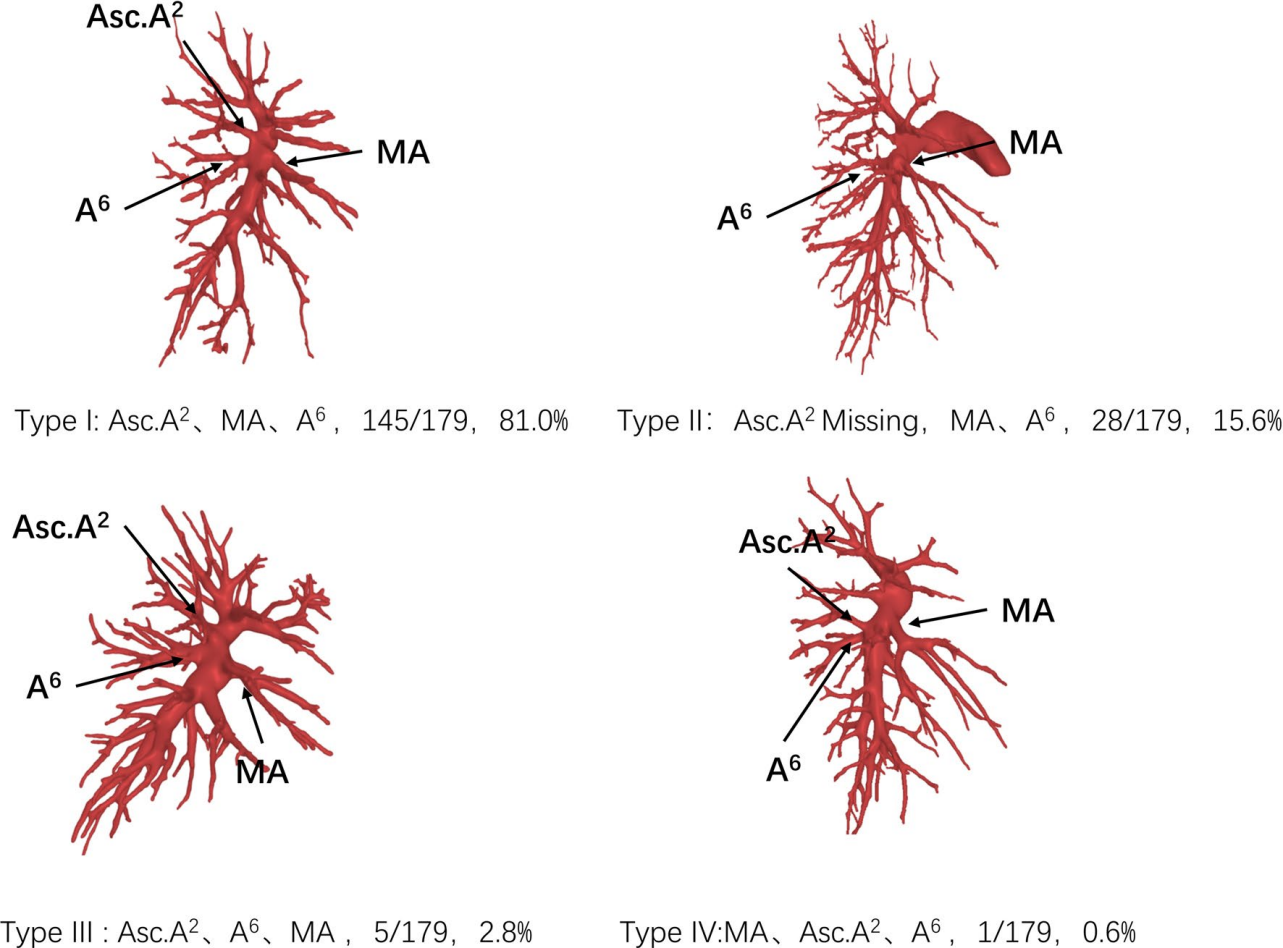
The RPA branching patterns of interlobar portion (RPA: right pulmonary arteries, MA: middle lobe artery, Asc.A2: posterior ascending artery)

**Fig. 4:**
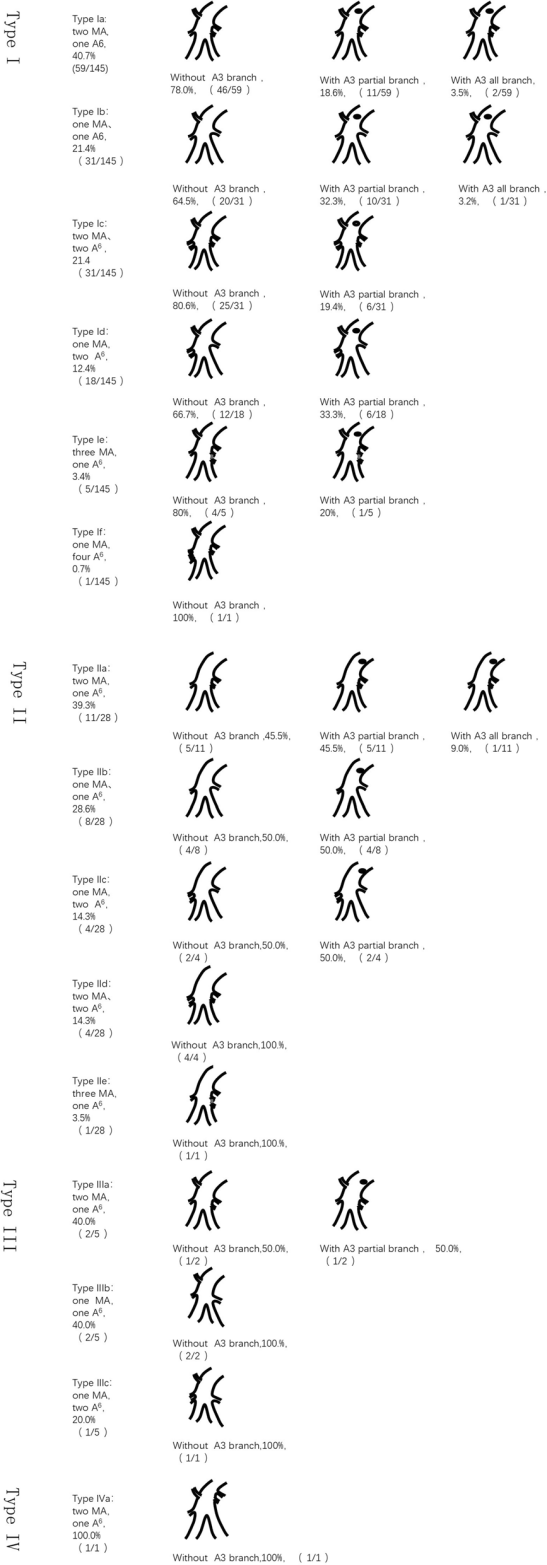
The RPA branching patterns of interlobar portion were classified into 15 subcategories according to the number of branches of MA and A6 (RPA: right pulmonary arteries, MA: middle lobe artery, Asc.A2: posterior ascending artery)

The RPA branching patterns of the interlobar portion were classified into 4 types according to the order and number of branches: Type I (145/179, 81.0%), Asc. A2, MA, A6; Type II (28/179, 15.6%), MA, A6 (Asc. A2 deletion); Type III (5/179, 2.8%), Asc. A2, A6, MA; and Type IV (1/179, 0.6%), MA, Asc. A2, A6 (Figs. 2, 3). The RPA branching patterns of interlobar portion were classified into 15 subcategories according to the number of branches of MA and A6. Type I contains 6 subcategories: Type Ia (59/145, 40.7%), two MA and one A6; Type Ib (31/145, 21.4%), one MA and one A6; Type Ic (31/145, 21.4%), two MA and two A6; Type Id (18/145, 12.4%), MA, two A6; Type Ie (5/145, 3.4%), three MA and one A6; and Type If (1/145, 0.7%), one MA and four A6 (Fig. 4); Type II contains five subcategories: Type IIa (11/28, 28.6%), two MA and one A6; Type IIb (8/28, 28.6%), one MA and one A6; Type IIc (4/28, 14.3%), one MA and two A6; Type IId (4/28, 14.3%), two MA and two A6; and Type IIe (1/28, 3.5%), three MA and one A6; Type III contains 3 subcategories: Type IIIa (2/5, 40%), two MA and one A6; Type IIIb (2/5, 40%), two MA and two A6; and Type IIIc (1/5, 20%), one MA and two A6; Type IV contains 1 subclass: Type IVa (1/1,100%), two MA and one A6 (Fig. 4).

Two MAs were the most frequent pattern (110/179, 61.5%), followed one MA (63/179, 35.2%), and three MAs constituted the least frequently observed pattern (6/179, 3.3%). One A6 branch was the most common type (118/179, 65.9%), followed by two branches (60/179, 33.5%), and only 1 patient had four A6 branches in our study. The anterior segment artery (A3) of the right upper lobe usually arises from the first branch of the main RPA. However, in this study, we found that 50 patients (27.9%) had several branches of A3 directly arising from the interlobar portion of the RPA. Among them, one or more branches of A3 originating from the interlobar portion were the most common type (37/50, 74.0%). In this study, we also observed that 41 patients (41/179, 22.9%) had Asc. A2 deletion and 27 patients (27/179, 15.1%) exhibited A*.

**Fig. 5:**
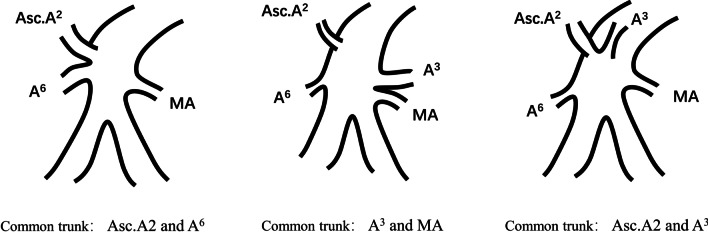
Common trunk of the RPA branching patterns of interlobar portion (RPA: right pulmonary arteries, MA: middle lobe artery, Asc.A2: posterior ascending artery)

We found that 3.4% of cases (n = 6) had a common trunk of Asc. A2 and apical artery of the lower lobe, and 1.1% of cases (n = 2) had a common trunk of branch of A3 and middle lobe artery. The rates observed in our study were in agreement with those in previous reports (2–9%, 1–4%) [12, 16]. We also detected a common trunk of Asc. A2 and A3 in 5.6% of cases (n = 10), and this type of common trunk had not been reported previously (Fig. 5).

## Discussion

Anatomical sublobar resection was previously applied in patients with weak pulmonary function, metastatic tumours or benign lesions. In addition, it is also a promising option for early-stage non-small cell lung cancer [17]. However, anatomical sublobar resection is complicated and requires a high level of surgical technique. Mastering pulmonary anatomy is of great importance for surgeons to complete surgery, especially segmentectomy/subsegmentectomy. Locating tumours where the pulmonary segment, bronchus, and blood vessels should be dissected is difficult for surgeons based only on axial images of chest CT scans. In recent years, 3D reconstruction has been widely applied because it accurately visualizes pulmonary anatomy, and the 3D reconstruction of pulmonary arteries (PA) has also been reported to be consistent with the types of PA branches [6, 18].

Higher blood pressure (22/8 mmHg, average pressure 13 mmHg), a thinner artery wall and a larger blood flow of the pulmonary artery increase the risk of pulmonary artery damage during surgery. Bleeding is more likely to be swift and violent and even causes death when pulmonary artery injury occurs [19]. Therefore, mastering the anatomy of the pulmonary artery is important for surgeons to complete surgery. Nagashima reviewed the pulmonary artery types of the right upper lobe in 263 patients based on three-dimensional reconstruction and classified the patterns into four types: superior trunk + Asc. A2 type (71.9%), superior trunk + inferior trunk + Asc. A2 type (13.7%), superior trunk (9.9%), and superior trunk + inferior trunk type (3.4%) [20]. However, this study did not provide a conclusion regarding the type and location of the middle lobe artery and A6. Fourdrain reported the anatomical types and proportions of the right pulmonary arteries by analysing 3D reconstructions of pulmonary angiography in 44 patients [12] but did not report the location of the vessels. The position of the interlobar portion of the RPA is particularly important during surgical resection, through which surgeons can distinguish branches and rare variations of pulmonary arteries.

In the present study, we diagrammatized the RPA branching patterns of the interlobar portion through thoracic thin-slice CT and three-dimensional reconstruction. Of all types, type I (Asc. A2, MA, A6) was the most common type (81.0%), and type IV (MA, Asc. A2, A6) was the least common type (0.6%). Further study shows that of all types, "two MA and one A6" is the most frequent pattern. Surgeons should give priority to the most common types during surgery rather than rare variant types (type III and type IV) and should also be distinguishing RPA branches to prevent intraoperative complications, such as vascular misdissection and injury. The data of this study showed that only 1 patient exhibited a type IV pattern; this patient had anatomical variation in B1 + 3 and B2 (such variants have been reported in previous cases [21]: B1 + 3 originates from the trachea, B2 originates from the right main bronchus). For this type, the branches of the A3 and MA should be carefully distinguished to avoid misdissection of the MA during anterior segmentectomy of the right upper lobe.

Our results showed that two branches of MA were the most common type (61.5%), and one branch was the less frequent type (35.2%). The least common type was three branches (3.3%), which was consistent with previous studies [12, 22]. We found a variation in one patient in the present study: the MA originated from the first branch of RPA and was accompanied by venous variation. The central vein was absent, and the middle lobe veins ascended between B5 + 6 and B1-3, drained into the inferior pulmonary vein, and finally drained into the right atrium. Surgeons should consider this variation to avoid injury of the MA when they plan to perform an apical segmentectomy or anterior segmentectomy. In addition, some studies have reported [11] that some branches of the MA can arise from the basal segment of the RPA, but we did not find this variation in our study. The most common type of A6 was one branch (65.9%), followed by two branches (33.5%). The least common type was four branches (0.6%), which has not been previously reported. We found one patient with one branch of A6 originating from A8 + 9 in our study. For patients with this variation, serious care should be taken during the resection of the superior segment of the lower lobe to avoid vessel branch injury and bleeding. A pattern of three branches of A6 was reported by Amore [11], but this pattern is rare (less than 1%); therefore, this variant type was not found in our study. In our study, we found that 41 patients (22.9%) had no Asc. A2, and all branches of A2 originated from the first branch of RPA. This rate was higher than that in a previous report (12–17%) [12], which may be related to racial differences. Surgeons did not need to separate branches of A2 originating from the interlobar portion of the RPA, which could not only reduce the operation time but also decrease the risk of A2 injury. Nomoru Yuaki described a prevailing pattern schema of A3 originating from the first branch of RPA. The data of this study showed that a total of 50 patients (27.9%) had several branches of A3 originating from the interlobar portion of RPA. In addition to the above types, 2 patients exhibited A3 variations, one branch of which originated from A5 (one of the branches of MA). This mutation should be carefully identified to avoid A3 injury during middle lobectomy and posterior segmentectomy.

Therefore, chest thin-slice CT and 3D reconstruction may help surgeons to distinguish the anatomical types of the pulmonary artery and rare vascular variations. We believe that the present RPA data and diagrammatized patterns will make a valuable contribution to the safety and ease of anatomical lobectomy and sublobectomy. Nevertheless, this study is subject to shortcomings: (1) It is a retrospective study and may have selection bias in the sample size. (2) The sample size was not sufficiently large, and some rare variant types may have been missed. A larger sample size is required to verify the accuracy of the results.

## Conclusions

Based on chest thin-slice CT scans and 3D reconstruction, our study is the first to describe the branching patterns and frequencies of the interlobar portion of RPA.Q3 3D reconstruction provides an understandable view of the anatomical types of pulmonary arteries and their variations. Chest thin-slice CT scans and 3D reconstruction play an essential role in VATS.

## Data Availability

The data and materials of this article are available.
